# Pubertal exposure to dietary advanced glycation end products disrupts ductal morphogenesis and induces atypical hyperplasia in the mammary gland

**DOI:** 10.1186/s13058-023-01714-4

**Published:** 2023-10-06

**Authors:** Bradley A. Krisanits, Reid Schuster, Jaime Randise, Lourdes M. Nogueira, Jackson T. Lane, Gowtami A. Panguluri, Hong Li, Kristi Helke, Maria C. Cuitiño, Christopher Koivisto, Laura Spruill, Michael C. Ostrowski, Steven M. Anderson, David P. Turner, Victoria J. Findlay

**Affiliations:** 1 Department of Pathology and Laboratory Medicine, Medical University of South Carolina, Charleston, SC USA; 2 Department of Public Health Sciences, Medical University of South Carolina, Charleston, SC USA; 3 Department of Comparative Medicine, Medical University of South Carolina, Charleston, SC USA; 4 Department of Biochemistry and Molecular Biology, Medical University of South Carolina, Charleston, SC USA; 5 Department of Pathology, University of Colorado Anschutz Medical Campus, Aurora, CO USA; 6 Present Address: Department of Surgery and Massey Cancer Center, Virginia Commonwealth University, Richmond, VA USA; 7 Present Address: Department of Public Health Sciences, University of California, Davis, CA USA; 8 Present Address: College of Health Sciences, Comprehensive Cancer Center, Ohio State University, Columbus, OH USA

**Keywords:** Advanced glycation end products, RAGE, Mammary gland development, Puberty, Diet, Microenvironment

## Abstract

**Background:**

Advanced glycation end products (AGEs) are reactive metabolites intrinsically linked with modern dietary patterns. Processed foods, and those high in sugar, protein and fat, often contain high levels of AGEs. Increased AGE levels are associated with increased breast cancer risk, however their significance has been largely overlooked due to a lack of direct cause-and-effect relationship.

**Methods:**

To address this knowledge gap, FVB/n mice were fed regular, low AGE, and high AGE diets from 3 weeks of age and mammary glands harvested during puberty (7 weeks) or adulthood (12 weeks and 7 months) to determine the effects upon mammary gland development. At endpoint mammary glands were harvested and assessed histologically (*n* ≥ 4). Immunohistochemistry and immunofluorescence were used to assess cellular proliferation and stromal fibroblast and macrophage recruitment. The Kruskal–Wallis test were used to compare continuous outcomes among groups. Mammary epithelial cell migration and invasion in response to AGE-mediated fibroblast activation was determined in two-compartment co-culture models. In vitro experiments were performed in triplicate. The nonparametric Wilcoxon rank sum test was used to compare differences between groups.

**Results:**

Histological analysis revealed the high AGE diet delayed ductal elongation, increased primary branching, as well as increased terminal end bud number and size. The high AGE diet also led to increased recruitment and proliferation of stromal cells to abnormal structures that persisted into adulthood. Atypical hyperplasia was observed in the high AGE fed mice. Ex vivo fibroblasts from mice fed dietary-AGEs retain an activated phenotype and promoted epithelial migration and invasion of non-transformed immortalized and tumor-derived mammary epithelial cells. Mechanistically, we found that the receptor for AGE (RAGE) is required for AGE-mediated increases in epithelial cell migration and invasion.

**Conclusions:**

We observed a disruption in mammary gland development when mice were fed a diet high in AGEs. Further, both epithelial and stromal cell populations were impacted by the high AGE diet in the mammary gland. Educational, interventional, and pharmacological strategies to reduce AGEs associated with diet may be viewed as novel disease preventive and/or therapeutic initiatives during puberty.

**Supplementary Information:**

The online version contains supplementary material available at 10.1186/s13058-023-01714-4.

## Background

There is a consensus that chronic diseases, including cancer, diabetes, cardiovascular and neurodegenerative diseases, are negatively associated with modern dietary patterns, especially those associated with high consumption of meat, sugar, oils and fats [1]. However, given the complexity of the various components of modern diets, there is less consensus regarding the contributions of specific dietary factors within the diet to specific health issues. The consumption of diets that are high in sugar and fats has clearly been associated with an increase in obesity and diabetes, both of which are associated with an increase in cardiac disease and cancer [2]. Increased alcohol consumption is also associated with numerous diseases including hepatic dysfunction. A less well appreciated dietary component that appears to contribute to disease and disease progression are advanced glycation end-products (AGEs). AGEs are reactive metabolites derived from sugars that are either produced endogenously as by-products of energy metabolism and oxidative stress, or can be acquired exogenously through dietary sources [3]. Specifically, AGE concentrations are increased 10 to100-fold in heat-treated food, especially fried or grilled foods that contain sugars [4]. In addition, AGEs are added to processed foods to increase stability and/or mouthfeel [4]. Because the consumption of processed foods, and subsequently AGE intake, has increased in modern times, it is important that we understand the potential for these compounds to alter development and lead to diseases.

AGEs are a group of complex biomolecules that are produced by the non-enzymatic glycation of biological macromolecules such as proteins, lipids, and nucleic acids. Reaction between activated carbonyl groups on sugars and amino groups on proteins, lipids and nucleic acids results in biomolecules with altered structure, function and bioactivity that can alter biological processes, cause cell damage, or induce cell death [5, 6]. Although AGEs can be produced intracellularly from sugars present in the diet (endogenous AGEs), they can also be present as preformed compounds in the diet that are added directly to processed foods (e.g. Nε-carboxy-methyl lysine (CML)), or they can be formed as a result of cooking or grilled foods (exogenous AGEs). Exogenous AGEs present in foods consist of a wide spectrum of low and high molecular weight compounds derived from multiple biological macromolecules that include proteins, lipids and nucleotides [7, 8]. To date, over forty types of AGE compound have been identified, classified largely upon their physical and structural properties as well as the actual molecule glycated [9]. Accumulation of AGEs in tissues and organs leads to their functional and physical degeneration, and may result in accelerated aging [5, 6]. Although AGEs can bind to scavenger receptors, the major receptor is the transmembrane receptor for AGE (RAGE) [10]. AGE/RAGE activation is a potent inducer of stromal-epithelial interactions which lead to persistent immune mediated inflammation and oxidative stress which in a feed forward loop lead to further AGE formation [11, 12]. In addition, the persistent nature of AGEs due to their inefficient removal from the body and the irreversible nature of many adducts, suggests they may be able to cause long-term disruption of developmental programs as well as normal tissue homeostasis. Due to modern dietary patterns, food is now a major source of AGE exposure with up to 30% of the AGEs consumed remaining in the body after consumption. Foods highest in AGEs include red and white meat, processed/manufactured foods and those high in sugar, protein and fat. The high dry heats and pressures applied during cooking (grilling, broiling, frying) and food manufacturing (extrusion, retorting, irradiation), drive non-enzymatic glycoxidation reactions in foods to rapidly increase AGE content. As noted above, manufacturers also add AGEs (e.g. Nε-carboxy-methyl lysine (CML)) directly to food to improve both taste and appearance [4–6, 13]. The bioavailability and mechanistic implications of dietary AGEs on disease phenotypes is largely undefined. While epidemiological studies consistently associate high AGE consumption with multiple disease phenotypes, basic and translational studies showing a direct cause and effect relationship are significantly lacking.

A role for dietary derived AGEs in disease phenotypes is supported by our recent collaborative population studies that positively correlate a high intake of dietary-AGE with breast cancer risk in both the ‘NIH-AARP Diet and Health Study’ and the ‘PLCO Cancer Screening Trial’ [14–16]. Despite this evidence and their role as drivers of pro-inflammatory and oxidative stressors, the significance of AGE content in foods has been largely overlooked due to a lack of basic experimental data that support a direct cause-and-effect relationship.

The mammary gland offers a unique opportunity to study the effects of diet and environmental factors upon both development and induction of disease as the majority of its development is post-natal thus allowing controlled analysis of dietary effects. Mammary gland development is one of the earliest signs of puberty and is characterized by extensive tissue remodeling driven by tightly regulated programs that engage both the epithelium and the extracellular matrix (ECM) [17–21]. Dysregulation of pubertal mammary development can impact adult mammary gland function as well as increase the risk of breast cancer in later life [19, 22, 23]. Nutritional intake as well as being overweight or obese has been shown to have a major impact on both the timing and regulation of pubertal development, and epidemiological studies have associated these factors with increased breast cancer risk [24–26]. Therefore, we have examined the effects of dietary AGEs upon mammary gland development in the mouse in order to gain insight into potential effects and mechanisms. To this end mice were provided a high sugar diet that would result in the endogenous production of AGEs, or alternatively provided a diet high in exogenous AGEs produced by autoclaving a high sugar diet to simulate production of AGEs by cooking [27]. Our data indicates the high AGE diet significantly alters mammary gland development within the pubertal window and suggest a need for further study using this model system.

## Methods

### Mouse model of dietary-AGE consumption

FVB/n mice (JAX stock #001800) were acquired from Jackson Labs. RAGE null (RAGE -/-) mice were a kind gift of Dr. B Arnold (German Cancer Research Center, Heidelberg, Germany) and were on the C57Bl6/J genetic background [28]. The RAGE null mice were backcrossed onto the FVB/n background through 8 generations. Genotyping was performed by Transnetyx using real-time PCR using primers to identify heterozygous and homozygous knockout mice.

All used in diet studies were generated in our breeding colony. Three week-old female mice were randomized and weaned to one of three diets: 1) regular mouse diet (LabDiet 5V75; St. Louis, MO); 2) low AGE diet (Envigo TD.98090; Cumberland, VA); 3) high AGE diet [Envigo TD.98090 that had been autoclaved 120 °C for 15 min to induce AGE formation [29]]. These diets consist of comparable kcal levels derived from fat, protein and carbohydrates, however they differ in the carbohydrate source; starch is the major carbohydrate present in the regular rodent chow, whereas glucose is the major carbohydrate in the two AGE-specific diets [29]. Although the two AGE-specific diets initially have the same glucose content, autoclaving the high AGE diet stimulates the formation of a wide spectrum of AGEs typically found in fried or grilled foods [8]. Our published studies illustrate the increased AGE levels in the diet after high temperature cooking (autoclaving) [27]. Litters of mice were divided randomly between different diet groups and multiple litters were used to generate the number of mice needed for each experiment. Diets were continued until endpoints of either seven weeks in our pubertal studies, 12 weeks in our mature virgin, or 7 months of age. Diet switch experiments were performed where mice were fed either the regular or high AGE diet from weeks 3–7, and then switched to either the high AGE [REG-HIGH] or regular diet [HIGH-REG] (respectively) from weeks 7–12. For wild type FVB/n mice: Regular diet (7 weeks *n* = 4; 12 weeks *n* = 4; 7 months *n* = 6), low AGE diet (7 weeks *n* = 5; 12 weeks *n* = 5), high AGE diet (7 weeks *n* = 5; 12 weeks *n* = 4; 7 months *n* = 6), switch diet (REG-HIGH diet *n* = 4; HIGH-REG *n* = 5). Weights of individual mice were determined weekly, and food consumption per cage recorded for longitudinal analysis. The average pup weight at 21 days was 13.05 ± 1.16 g with a range of 11.6 – 14.4 g. At the study endpoints, inguinal mammary glands were extracted from the mice and either whole-mounted, or fixed in 10% neutral-buffered formalin and then paraffin embedded for immunohistochemistry (IHC) and hematoxylin and eosin (H&E) staining. Blood was collected at each endpoint via cardiac puncture, as well as a subset of mice at 3 weeks for baseline measurements. Estrous staging of all mice was confirmed by two independent pathologists using established protocols [30]. Mice in diestrus were excluded from any analyses involving proliferation (see below and in text). To minimize potential confounders, all mice were housed in the same location, all diets were administered at the same time to mice of the same age, and both water and food were provided ad libitum. All mice were weighed at the same time.

### Mouse tissue analysis

Tissues were fixed in neutral buffered formalin (NBF)-fixed and paraffin-embedded. Mouse mammary gland and uterus tissue were sectioned (5 µm), deparaffinized, rehydrated and stained via conventional H&E. Histological assessment of ductal and TEB morphology was performed by two independent pathologists. IHC staining for Ki67 (Lab Vision™ RM9106, 1:100) and SMA (Invitrogen PA5-18,292; 1:100) were performed as previously described [31]. For staining with F4/80 (eBioscience™ 14–4801–82; 1:100) and Vimentin (Abcam ab345939; 1:100), samples were processed in the MUSC Translational Science Laboratory using the Ventana Discovery Ultra automated stainer. Imaging was completed using an Echo Rebel microscope (Echo, San Diego, CA). Quantification of IHC staining was performed using Echo software. Spectral analysis was performed by the Human Immune Monitoring Shared Resource (RRID:SCR_021985) within the University of Colorado Human Immunology and Immunotherapy Initiative and the University of Colorado Cancer Center (P30CA046934) to assess macrophage polarization.

### Hormone and rage ELISA assays

Serum Estradiol and Progesterone levels were determined using ELISA kits from Cayman Chemical (Ann Arbor, MI; kit #501890 and #582601, respectively). Mouse RAGE levels were determined using ELISA kits from R&D Systems (Minneapolis, MN; #DY1179) and performed as per the manufacturers’ instructions.

### Whole mount staining and quantification

Isolated mammary glands were stained overnight in carmine staining solution (Sigma-Aldrich: 0.2% carmine, 0.5% aluminum potassium sulfate) at room temperature and then processed for preparation of whole mounts by standard methodology [32]. TEB number was counted across the entire width at the distal edge of the mammary gland and reported as number per gland. TEB area, branch points, and ductal extension were quantified using digital Echo software (Echo, San Diego, CA).

### Cell lines and culture conditions

HC11 cells are immortalized, non-transformed mammary epithelial cells of mouse origin [33] and were purchased from ATCC (Manassas, VA). Cells were incubated at 37 °C, 5% CO2 in RPMI media containing 10% fetal bovine serum (FBS), 1% Penicillin/Streptomycin (P/S), 2 μg/mL insulin (Invitrogen) and 10 ngη/mL Epidermal Growth Factor. Met1 tumor cells were derived from mouse mammary gland carcinoma that arose in a transgenic MMTV-PyMT (mouse mammary tumor virus-polyoma middle tumor-antigen) mouse and this cell line has been used to model triple negative breast cancer [34]. The Met1 cell line was obtained from Alexander Borowsky (University of California Davis Comprehensive Cancer Center) and maintained in Dulbecco’s modified Eagle’s medium (DMEM) containing 10% FBS and 1% P/S. All media components were obtained from Invitrogen.

Primary fibroblasts were isolated from the abdominal gland of 7 week old FVB/n, RAGE +/+ and RAGE-/- mice fed either a regular or high AGE diet. Mammary glands were removed aseptically, minced and incubated in digestion solution (F12 media + 5% FBS containing 3 mg/mL collagenase type 3 (Worthington Biochemical Corporation) for 1 h at 37 °C with occasional vortexing. After sitting at RT for 10 min the cell solution was centrifuged for 5 min at 2500 RPM to remove apidocytes. The supernatant was collected, centrifuged at 500 g for 5 min and the pellet re-suspended in F12. The resuspension was filtered through a 40 μm mesh, centrifuged at 500 g for 5 min and plated in F12 plating media (Hams F12, 5% FBS, 1% P/S, 10 ng/mL EGF, 10 μg/mL insulin, 2 μg/mL hydrocortisone). Primary fibroblasts were TERT immortalized using the hTERT Antigen Cell Immortalization Kit (Cat #CILV02) from ALSTEM (Richmons, CA) and performed according to the manufacturers’ instructions.

### qPCR analysis

Total RNA from fibroblasts was extracted using the RNeasyPlus Mini Kit (Qiagen; Valencia, CA). 1 μg total RNA was reverse transcribed in a 20 μl reaction using iScript (Bio-Rad; Hercules, CA). Real time PCR for gene expression was performed with 5 μl of a 1:20 dilution of reverse transcribed cDNA using the universal probe library (UPL) system (Roche, Nutley, NJ) in a LightCycler 480 (Roche, Nutley, NJ). The cycling conditions were performed as per the manufacturer's instructions. Triplicate reactions were run for each cDNA sample. The relative expression of each gene was quantified on the basis of Ct value measured against an internal standard curve for each specific set of primers using the software provided by the instrument manufacturer (Roche, Nutley, NJ). These data were normalized to TBP. Primer sequences and probe numbers are provided in Table 1. RAGE primers and probe were commercially available and purchased from BioRad.

**Table 1:** Primers and probes used in the study

Gene	UPL Number	5’ primer sequence	3 primer sequence	Amplicon length
COL1A1	21	cagggtcctcctggttctc	gaccgttgagtccgtctttg	124 nt
FSP1	56	ggagctgcctagcttcctg	tcctggaagtcaacttcattgtc	102 nt
FAP	71	tggctgaaaactgtctttgga	ctttgtgtttccttcaggtttgt	108 nt
TBP	11	ggggagctgtgatgtgaagt	ccaggaaataattctggctcat	93 nt
IGF1	104	gaagcctacaaaagcagccc	tagggacggggacttctgag	79 nt
RANKL	78	ctgcaacacattgtggggc	ccacatccaaccatgagcct	73 nt
AREG	73	aagaaaacgggactgtgcat	ggcttggcaatgattcaact	73 nt

### Migration and invasion assays

Migration of epithelial cells was measured using 8.0 μM Transwell inserts coated in diluted fibronectin (100 μg/mL) and placed into 12-well plates (VWR). For migration assays, either HC11 or Met1 (5 × 10^4^) epithelial cells in 0.5 mL serum-free media were placed in Transwell coated inserts, and placed over a bottom well that either contained or lacked 5 × 10^4^ immortalized fibroblasts plated in 0.5 mL full serum media in the bottom. In some studies, 50 μg/mL BSA-AGE (EMD Millipore; Cat #121800) was added to the top or bottom compartment. Invasion assays were performed as described above with the following changes. Transwell inserts were coated with Cultrex® Basement Membrane Matrix (BME) using a kit from Trevigen (Gaithersburg, MD) and placed in 24 well plates. Cells (2.5 × 10^4^) were placed in the upper and lower chambers. Cells were incubated for 24 h. Cells were stained using a Kwick-diff stain kit from Thermo Fisher. All assays were performed with experimental duplicates and with biological triplicates. Migrated cells were quantified by counting total number of cells per field of view in a total of five fields per insert using Echo software. Average number of cells per field is reported.

### AGE assessment

AGE levels in pre and post-diet serum samples was evaluated using OxiSelect™ AGE Competitive ELISA Kit (Cell Biolabs, San Diego, CA) as per the manufacturer’s instructions.

### Statistical analysis

Values are presented as means ± SEM for in vivo and ± SD for in vitro experiments respectively. For in vivo studies, data were analyzed for main effects of diet and age. Continuous outcomes were measured for different sets of animals at different time points. No other covariates were measured. Number of mice in some analyses varied owing to the randomness of sex in pup litters for diet assignments at weaning and estrous stage. Therefore, due to small sample sizes, the Kruskal–Wallis test were used to compare continuous outcomes among groups. If a significant result was discovered between diet and age, all possible pairwise comparisons were performed. For in vitro and ex vivo studies, the nonparametric Wilcoxon rank sum test was used to determine statistical significance using GraphPad. *P* < 0.05 was considered statistically significant.

## Results

### Consumption of a high AGE diet results in altered development of the mammary ductal tree

To investigate the effects of dietary AGEs on pubertal mammary gland development, wild type FVB/n mice were fed regular (control), low AGE and high AGE diets ad libitum starting at three weeks of age. The high AGE diet was produced by autoclaving the low AGE diet to simulate the sugar driven production of AGEs by cooking. As previously observed in other studies [35, 36] we did not observe significant differences in body mass or consumption of chow between mice provided with the different diets over the course of the 12-week study (Additional file 1: Figure s1). Increased AGE levels were confirmed in the serum of mice fed the high AGE diet for four weeks (at seven weeks of age) (Additional file 1: Figure s1). It should be noted that this level of serum AGEs is comparable to that observed in breast cancer patients [37].

**Fig. 1:**
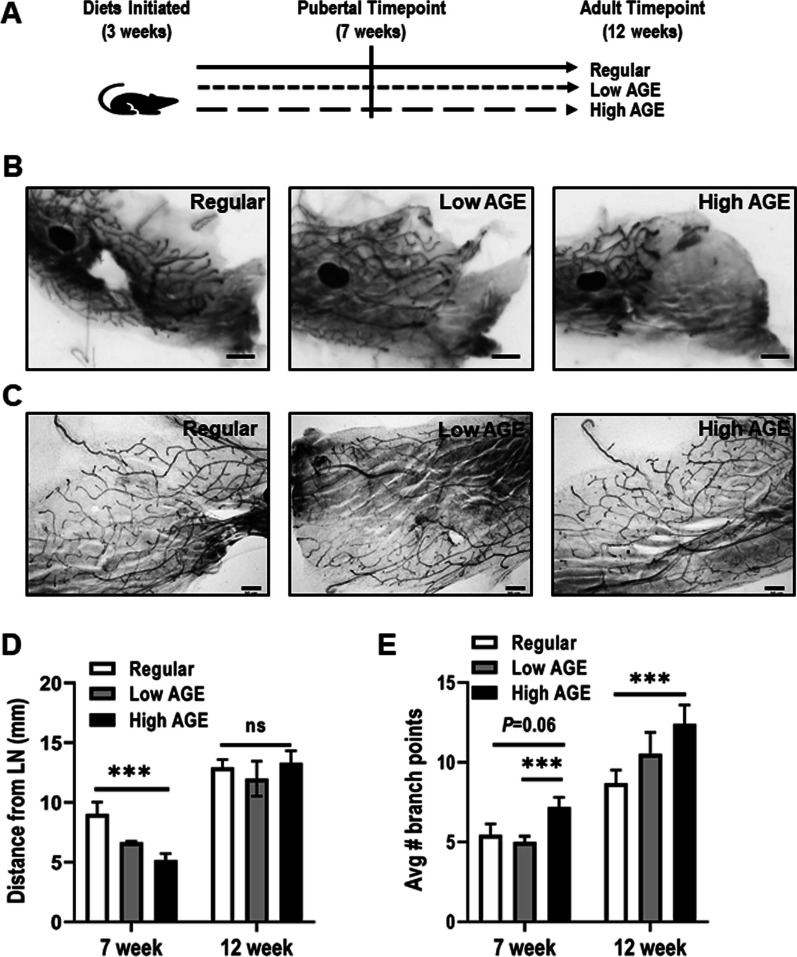
Consumption of a high AGE diet results in disorganization of the mouse mammary ductal tree. **A** Schematic representation of experimental design for examining the effect of AGEs upon mammary gland development. **B** Representative images of carmine stained whole mounts from 7- **B** and 12- **C** week old mice fed regular, low AGE or high AGE diet. **D** Quantification of ductal extension beyond the central lymph node in 7-week old mice. **E** Quantification of branch points in 7- and 12-week old mice. Scale bar = 2 mm (**B**) and 50 µM (**C**). Mouse numbers: 7 week—regular (*n* = 4), low AGE (*n* = 5), high AGE (*n* = 5); 12 week—regular (*n* = 4), low AGE (*n* = 4), high AGE (*n* = 4). Values are mean ± SEM. ***P < 0.05

The effects of AGEs upon the architecture of the mammary ductal tree was examined using histo-morphological analysis of whole mounted mammary glands at seven and twelve weeks of age corresponding to puberty and adulthood, respectively (Fig. 1A). A rudimentary ductal structure is present at birth and during puberty ducts elongate from this structure and grow past the central lymph node to the distal end of the inguinal mammary gland of the mouse [38]. Thus, the lymph node can be used as a landmark for ductal elongation, and generally ducts do not extend past the lymph node until after five weeks of age in the FVB/n strain [38]. As can be readily accessed from the whole mounts, ductal elongation is diminished in mice provided the high AGE diet compared to those provided a regular diet, with mice provided the low AGE diet having an intermediate phenotype (Fig. 1B). As measured by the distance travelled from the lymph node to the tip of the furthest TEB in the leading edge, there was a significant reduction in ductal extension in mice fed a high AGE diet when compared to regular fed mice (Fig. 1B, D). The reduction in ductal elongation in mice provided the high AGE diet was transient as no significant differences were observed between mice provided the different diets at the 12 week timepoint when the ductal tree is fully developed (Fig. 1D).

Ductal branching also occurs during puberty resulting in a complete mammary tree that fills the mammary fat pad; therefore, we assessed the effects of dietary-AGE on ductal branching by quantitating the number of branch points from the primary branch. High AGE consumption significantly increased the number of branch points at both the 7- and 12-week time points compared to both regular and low AGE diet groups (Fig. 1C & E). The development of the pubertal mammary gland is controlled by hormonal regulation at the systemic level, particularly estrogen during puberty [39]. To determine if changes in systemic hormones were contributing to the observed AGE mediated effects on pubertal development, we performed ELISA assays for estradiol and progesterone in serum from mice at both 7- and 12-weeks of age. There were no significant differences in the circulating levels of estradiol and progesterone in mice provided the high AGE diet versus other experimental groups indicating that dietary-AGEs did not alter mammary development directly through these hormonal mediators (Additional file 2: Figure s2). We also performed qPCR for AREG, RANKL and IGF1 in the 7 week old mice and observed a significant increase in the levels of IGF1 (Additional file 2: Figure s2), suggesting that AGE-induced changes in the mammary gland may be mediated by pituitary hormones.

### Consumption of a high AGE diet results in abnormal terminal end bud morphology and proliferation

**Fig. 2:**
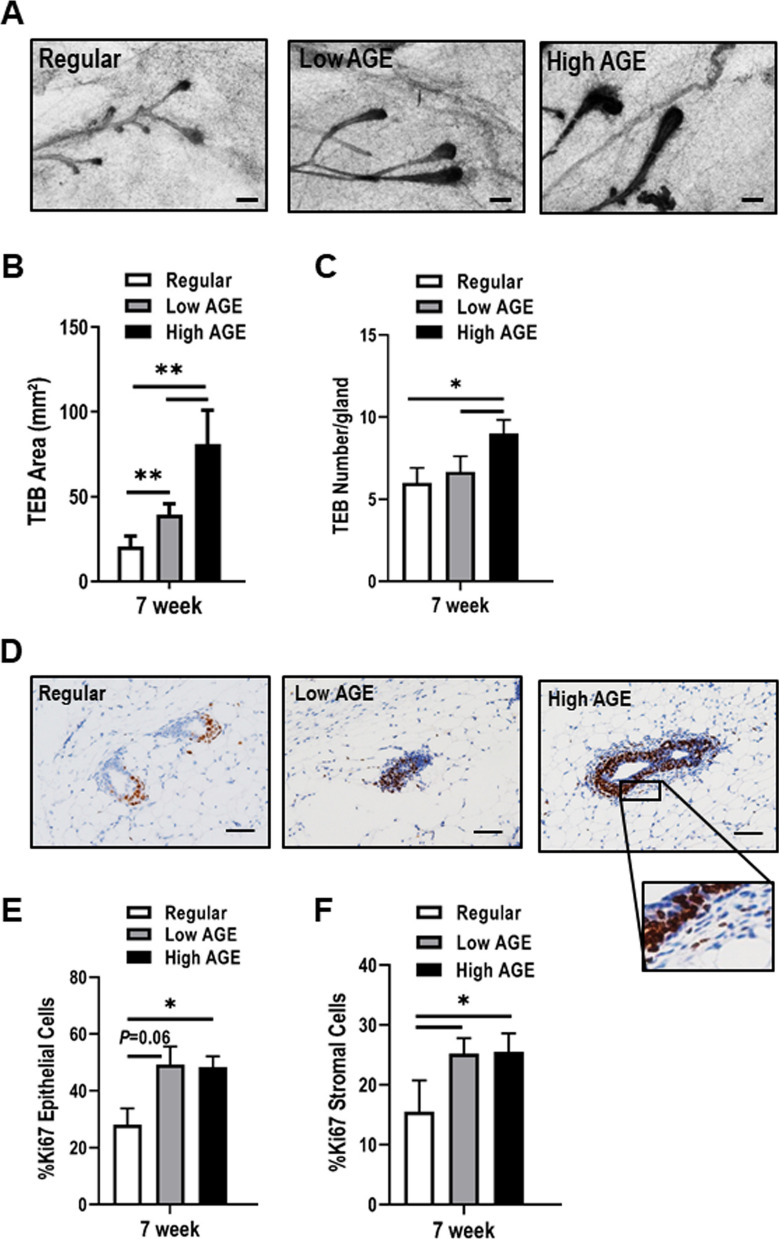
Consumption of a high AGE diet results in abnormal TEB morphology and proliferation. **A** Representative carmine stained whole mount images of TEBs from 7-week old mice fed regular, low AGE or high AGE diet. Scale bar = 50 µM. Quantification of TEB size (**B**) and TEB number (**C**) in 7-week old mice fed regular, low AGE and high AGE diets. Mouse numbers (**B**-**C**): regular (*n* = 4), low AGE (*n* = 5), high AGE (*n* = 5). **D** IHC staining of Ki67 in TEBs from 7-week old mice fed a regular, low AGE and high AGE diet. Inset shows proliferating cells in the stroma surrounding the TEBs in the high AGE fed mice. Scale bar = 50 µM. **E** Quantitation of Ki67 in TEBs of 7-week old mice fed regular, low AGE and high AGE diet. **F** Quantitation of Ki67 in the stromal cells surrounding TEBs of 7-week old mice fed regular, low AGE and high AGE diet. Mouse numbers (**D-F**)**:** regular (*n* = 3), low AGE (*n* = 4), high AGE (*n* = 4). Values are mean ± SEM. *P < 0.05; **P < 0.01

Terminal end buds (TEBs) are light bulb-shaped structures in which the more undifferentiated and more proliferative cells are located in the bulb region and the more differentiated, less proliferative cells are located in the neck region and the connecting duct [23]. We first examined TEBs in carmine stained whole-mount mammary glands from mice provided the various diets and observed larger TEBs in mice provided the high AGE diet (Fig. 2A). Quantitative assessment of the whole mounts demonstrated there was a significant increase in TEB size (area) in mice fed the high AGE diet compared to mice provided either the regular or low AGE diets at 7 weeks (Fig. 2B). There was also a significant increase in the size of TEBs in mice fed the low AGE diet when compared to the regular diet (Fig. 2B). Quantitation of TEB number revealed a significant increase in TEB number in mice fed the high AGE diet at 7 weeks compared to mice provided the regular and low AGE diets (Fig. 2C). The increase in TEB number was not observed in the mice fed the low AGE diet.

We next assessed TEB proliferation and observed significant head to tail polarity with Ki67 staining at the leading edge of the TEB in pubertal mammary tissue extracted from regular fed mice as expected (Fig. 2D). However, in the low AGE and high AGE fed mice, proliferation was observed throughout the TEB with no defined head to tail orientation of proliferation, indicating a disruption of defined TEB polarity (Fig. 2D). Quantification of Ki67 showed a significant increase in epithelial cell proliferation in the TEB structures from high AGE fed mice compared to that observed in regular fed mice (Fig. 2E). The proliferation in the low AGE fed mice showed a non-significant trend when compared to the regular fed mice (Fig. 2E). Proliferation of recruited stromal cells surrounding the TEB structures was also observed in mice fed the high AGE diet (Fig. 2D—inset). Quantification of stromal proliferation showed that the mice fed the low and high AGE diets had significantly increased proliferative stromal cells surrounding TEB structures when compared to the regular diet (Fig. 2F).

### High AGE consumption results in increased stromal recruitment to TEB structures

**Fig. 3:**
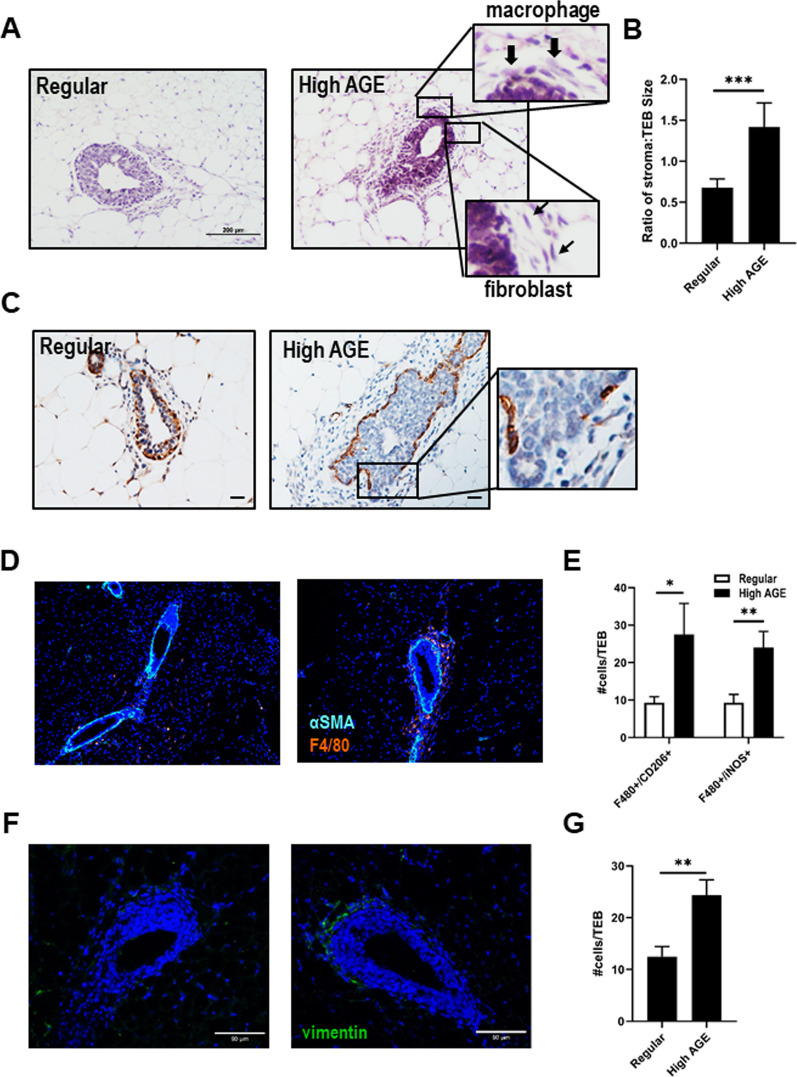
High AGE consumption results in increased stromal recruitment to TEB structures. **A** Representative H&E images of TEB structures from 7-week old mice fed a regular or high AGE diet. Inset shows increased magnification of histologically identified and recruitment of macrophages (arrowheads) and fibroblasts (arrow) around TEB structure. Scale bar = 200 µM. **B** Quantitation of the ratio of recruited stroma to TEB structure in 7-week old mice fed a regular and high AGE diet. **C** IHC staining of α-SMA (myoepithelial cells) in mice fed a regular or high AGE diet. Scale bar = 20 µM. Inset shows increased magnification of break in myoepithelial cell layer. **D-G** Representative immunofluorescent images and quantitation of F4/80 (**D/E**) and vimentin (**F/G**) surrounding TEB structures in 7 week old mice fed a regular or high AGE diet. Mouse numbers: 7 week—regular (*n* = 4), low AGE (*n* = 5), high AGE (*n* = 5). Values are mean ± SEM. *P < 0.05; **P < 0.01; ***P < 0.005

TEBs in mice provided the different diets were further characterized by examination of hematoxylin and eosin-stained thin sections to determine the structure, cellularity, and the identity of cells present within these structures. We observed structures typical of TEBs in mice provided a regular diet; a club-like structure composed of 4–6 cell layers and the leading edge was noted to contain few cells in the surrounding area and no stroma, although a surrounding stroma was evident at the distal end (Fig. 3A). This structure contained a layer of myoepithelial cells that stained positive for alpha smooth muscle actin (αSMA; Fig. 3C). In contrast, the TEBs observed in mice provided the high AGE diet were significantly different (frequency of abnormality ~ 73%) in that they were surrounded by extensive extracellular matrix (ECM) containing greater cellularity (Fig. 3A). We observed by histology that the ECM surrounding TEBs was increased in the mice fed the high AGE diet. The ECM appeared thicker, less localized to the neck and instead fully surrounded the TEB structure in the high AGE fed mice (Fig. 3A). The size of the TEB structure and surrounding stroma were quantified and we observed that the ratio of recruited stroma to TEB size was further increased in the mice fed the high AGE diet (Fig. 3B).

Further microscopic examination revealed that the predominant cell types within the abnormal ECM were fibroblasts and macrophages (Fig. 3A—insets). Immunofluorescent (IF) staining with F4/80 and vimentin confirmed the increased recruitment of these cell types within the ECM to the TEB structures in mice fed the high AGE diet when compared to regular control diet (Fig. 3D-G). Spectral analysis of macrophage polarization markers CD206 and iNOS further showed that although the total number of M1 and M2 macrophages surrounding TEBs was significantly increased the ratio of M1:M2 polarization remained the same (Fig. 3E).

The myoepithelial layer surrounding ducts and TEBs influences epithelial/stromal crosstalk and serves as a critical barrier for tumor epithelial cells. An examination of the myoepithelial layer using αSMA demonstrated significant breaks in this layer in the TEBs examined in mice fed a high AGE diet when compared to the intact myoepithelial layer observed in regular fed mice (Fig. 3C—inset). Breaks in the myoepithelial layer of at least one TEB per gland was observed in each of the mice fed a high AGE diet.

### High AGE consumption results in abnormal ductal morphology

**Fig. 4:**
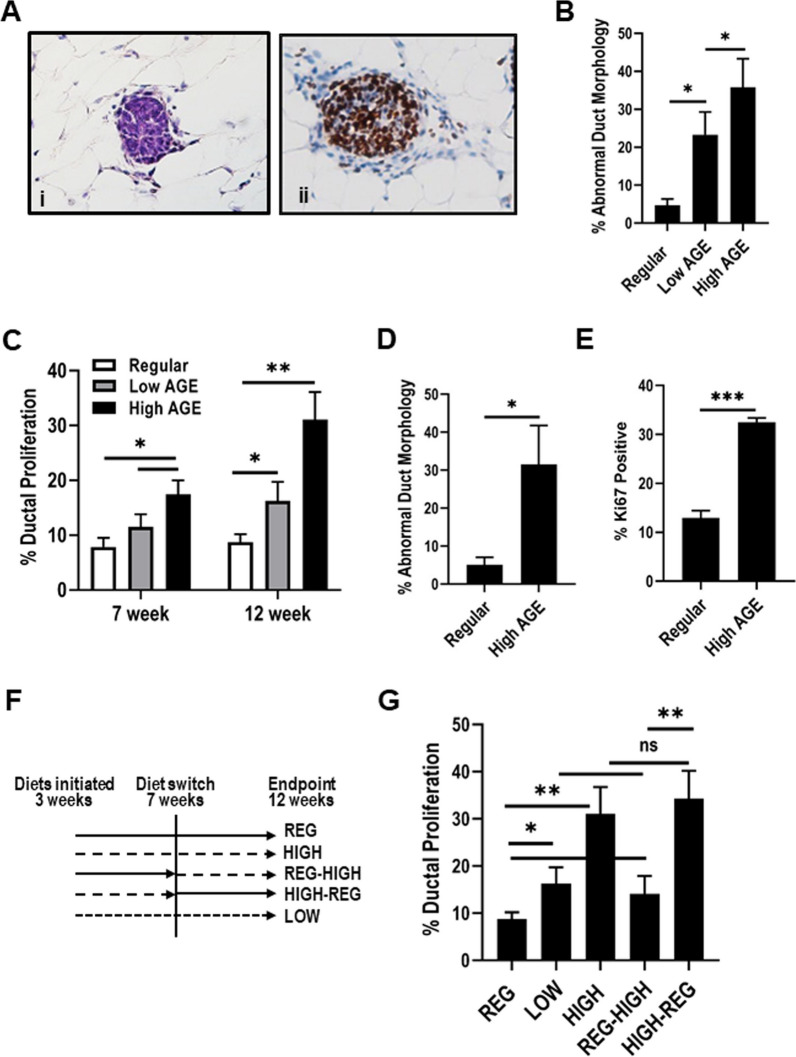
High AGE consumption induces atypical ductal hyperplasia. **A** Representative images of ducts representing low grade MIN from 12-week old mice fed a high AGE diet stained with H&E (i) and Ki67 (ii). **B** Quantitation of the percentage of abnormal ducts observed within the 12-week mammary glands of mice fed the different diets. (**C**) Quantitation of Ki67 in ducts of 7- and 12-week old mice fed regular, low AGE and high AGE diet. Quantitation of the percentage of (**D**) abnormal ducts and (**E**) Ki67 observed within the mammary glands of 7-month old mice fed the regular and high AGE diet. **F** Schematic representation of experimental design for AGE diet switch experiments on mammary gland development. **G** Quantitation of Ki67 in ducts of 12-week old mice fed switch diets. Mouse numbers: 7 week—regular (*n* = 3), low AGE (*n* = 4), high AGE (*n* = 4); 12 week—regular (*n* = 3), low AGE (*n* = 4), high AGE (*n* = 3) REG-HIGH (*n* = 4), HIGH-REG (*n* = 5); 7 month – regular (*n* = 3); high AGE (*n* = 4). Values are mean ± SEM. *P < 0.05; **P < 0.01; ***P < 0.005

We next examined the effects of chronic AGE consumption on mammary ductal structures. Histological assessment by a pathologist of the mature (12 week) mammary glands from mice fed a high AGE diet showed an array of abnormal ductal structures including atypical hyperplasia (Additional file 3: Figure s3) and what appeared to be mammary intraepithelial neoplasia (MIN) (Fig. 4A). Hyperproliferation was confirmed in the abnormal structures as evidenced by increased epithelial Ki67 staining, as well as increased and proliferative stroma (Fig. 4Aii). To determine if there was a significant difference in the percent of ducts with abnormal morphology between the different diets (and to account for abnormalities as a result of the angle of tissue slicing) we quantified abnormal ductal morphology. We found that both the low and high AGE-fed mice had significantly increased percentage of abnormal ducts when compared to mice provided the regular diet (Fig. 4B). To examine if the abnormal ductal structures were in one specific area of the gland we assessed their distribution by dividing the mammary gland into a proximal region that contained the nipple, middle region, and distal region. We observed abnormal ductal structures throughout the mammary gland and although there was a trend towards the distal location in mice fed the low and high AGE diets, no significant differences were observed (Additional file 3: Figure s3).

When the number of Ki67-positive cells in ductal structures is quantitated in mice of different ages, we observed relatively uniform pattern of proliferation in ducts in mice fed the regular diet through puberty and into adulthood, whereas mice fed the low and high AGE diet had significantly increased numbers of Ki67-postive cells, with the highest numbers being observed in mice fed the high AGE diet at 12 weeks (Fig. 4C). Significantly, the abnormal ductal morphology phenotype and the increased ductal proliferation was sustained at comparable levels in 7-month old mice continuously fed the high AGE diet (Fig. 4D, E).

### Dietary-AGE mediated effects on mammary morphogenesis are sensitive to the timing of pubertal exposure

To determine if the timing of exposure to the high AGE diet had an effect on mammary morphogenesis we performed a diet switch study in which one group of mice were initially fed a high AGE diet during pubertal growth (3–7 weeks) and then switched to a regular diet as it approaches adult maturity (8–12 weeks) [HIGH-REG]. Conversely, a second group of mice were initially fed a regular diet during pubertal growth 3–7 weeks), then switched to a high AGE diet as they approach maturity (8–12 weeks) [REG-HIGH] (Fig. 4F). An assessment of ductal proliferation at the 12-week time point was used as a read-out to assess the effects of the timing of high AGE diet upon ductal development when compared to mice continually fed the regular and high AGE diets. Consistent with our previous studies, an increased in ductal proliferation was observed in mice provided either the low or the high AGE diet compared to mice provided the regular diet with the highest number being observed in high AGE fed mice (Fig. 4G). We found that exposure to a high AGE diet at any time during the study resulted in an increase in ductal proliferation when compared to mice provided a regular diet, however, the greatest effect was observed when mice were exposed to the high AGE diet during the 3 to 7-week time period (Fig. 4G). In fact, ductal proliferation in the HIGH-REG group of mice was not statistically different from that observed in mice continuously provided a high AGE diet (Fig. 4G). This suggests that although AGEs may have an effect upon tissue at any time; specific tissues may have windows of more extreme sensitivity.

### High AGE consumption stimulates fibroblasts to promote cellular migration and invasion

**Fig. 5:**
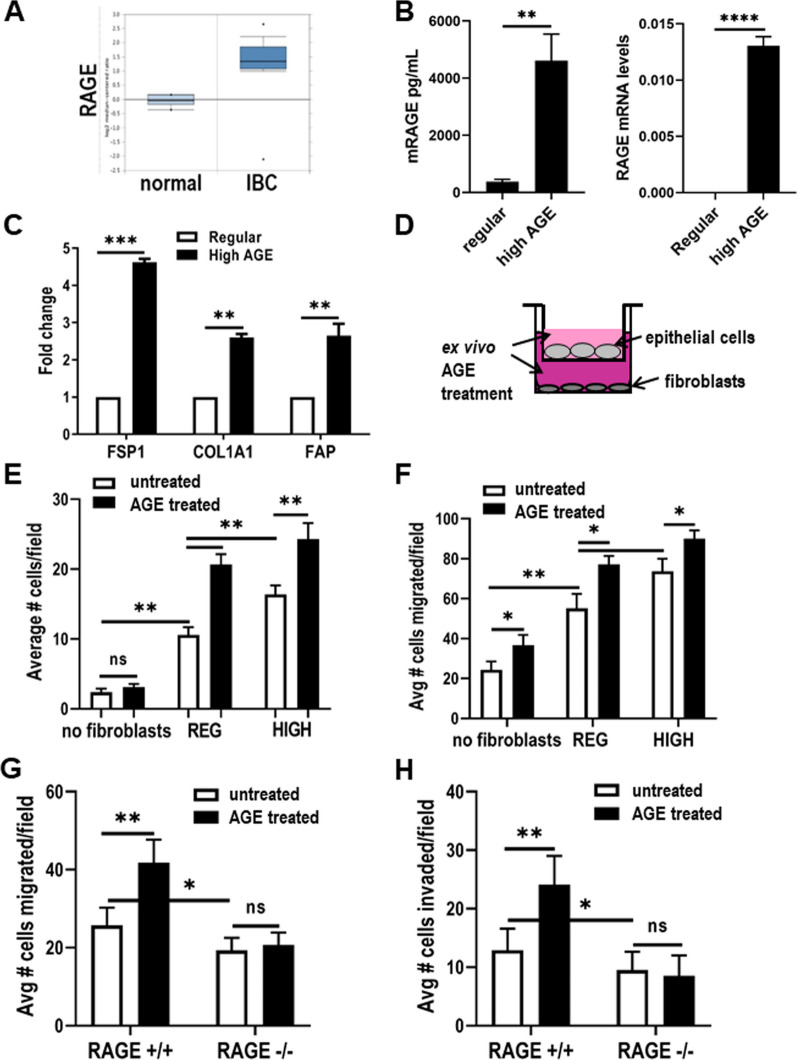
AGE stimulates fibroblasts to promote cellular migration and invasion in a RAGE dependent manner. **A** RAGE mRNA stromal levels in normal and invasive breast cancer cases from Oncomine. **B** ELISA (left panel) and qPCR (right panel) analysis of RAGE levels in primary mouse mammary fibroblasts isolated from 7-week old mice fed regular or high AGE diet. **C** qPCR analysis of mouse mammary fibroblasts isolated from 7-week old mice fed regular or high AGE diet. **D** Schematic representation of two compartment transwell co-culture assay. Transwell migration assay of HC11 **E** and Met1 **F** cells co-cultured with either no fibroblasts or fibroblasts isolated from mice fed a regular (REG) or high AGE (HIGH) diet. Fibroblasts in the lower chamber were also either untreated or treated with AGE ex vivo. Transwell migration (**G**) and invasion (**H**) of Met1 epithelial cells co-cultured with fibroblasts isolated from RAGE +/+ (regular *n* = 2; high AGE *n* = 2) or RAGE-/- (regular *n* = 2; high AGE *n* = 2) mice, and either untreated or treated with ex vivo AGE. Technical Replicates (*n* = 2); Biological Replicates (*n* = 3). Values are mean ± SD. *P < 0.05; **P < 0.01; ***P < 0.005; ****P < 0.001

Gene expression studies of the developing mammary gland have not observed the expression of RAGE in mouse mammary epithelial cells [40], leading us to question which cell types present in the mammary gland could be responsible for the observed effects of the high AGE diet. Several studies have described increased levels of RAGE in breast tissues from breast cancer patients compared to non-cancer patients. However, there have been no specific studies examining RAGE expression in the stromal cell compartment. We performed data mining in Oncomine [41] and found that RAGE mRNA levels are significantly elevated (*P* value 3.25^–12^) in stroma from invasive breast cancer patients when compared to normal breast stroma (Fig. 5A) [42]. Because mouse mammary tissue as a whole is reported to not express RAGE [40], we used ELISA and qPCR analysis to determine whether primary fibroblasts isolated from mouse mammary gland expressed RAGE. We found that mouse mammary fibroblasts do express RAGE, and that increased mRNA and protein RAGE levels are observed in fibroblasts isolated from mice fed a high AGE diet (Fig. 5B). Since the majority of the pathologic effects of AGEs are thought to be mediated through activation of RAGE [43], we hypothesized that stromal fibroblasts could be the mediator of the effects of the high AGE diet described above.

As a first means to determine whether fibroblasts are modified in mice provided a high AGE diet, we examined the expression of a panel of markers for fibroblast activation. Primary fibroblasts were isolated from 7-week-old mice fed either a regular or high AGE diet from three weeks of age. Quantitative gene expression analysis revealed the increased expression of ferroptosis suppressor protein 1 (FSP1), alpha-1 subunit of the fibril-forming type I collagen (COL1A1), and fibroblast activation protein (FAP), suggesting that the high AGE diet resulted in fibroblasts assuming the phenotype of activated fibroblasts similar to that found in cancer-associated fibroblasts (Fig. 5C) [44].

We next examined the effects of these primary fibroblasts on migration of either normal (HC11) or tumor (Met1) breast epithelial cells using ex vivo two compartment co-culture (Transwell) models (Fig. 5D). We observed the increased migration of HC11 cells towards fibroblasts when compared to culture alone. A significant increase in migration was also observed when HC11 cells were cultured with fibroblasts isolated from mice fed a regular diet when compared to fibroblasts isolated from mice fed a high AGE diet (Fig. 5E, white bars). Addition of exogenous AGE to the media in the fibroblast-containing lower chamber further stimulated HC11 cell migration (Fig. 5E, black bars). Because HC11 cells represent “normal” mammary epithelial cells, these studies were repeated with Met1, an immortalized mouse mammary tumor-derived cell line [34]. Although the basal level of Met1 migration without fibroblasts was higher, we observed a consistency with data obtained with HC11 cells, that migration of Met1 was increased when co-cultured with fibroblasts from mice provided a high AGE diet (Fig. 5F, white bars), and treatment of fibroblasts with exogenous AGEs further stimulated epithelial cell migration (Fig. 5F, black bars). We also examined the impact of AGE exposed fibroblasts upon the invasive potential of Met1 cells co-cultured with REG and HIGH fibroblasts and observed similar trends to that observed for epithelial cell migration (Additional file 4: Figure s4). The dietary effects of a high AGE diet induces a stable change in the mammary fibroblasts as HIGH fibroblasts were able to stimulate enhanced epithelial cell migration and invasion even after multiple passages in culture media lacking exogenous AGE, suggesting a sustained effect of the in vivo exposure of AGE on the fibroblasts. The effect of exogenous AGE treatment was also assessed when added directly to the epithelial cells in the upper chamber of the epithelial migration assay. We found that the addition of AGEs to the epithelial cells directly had no effect on non-transformed HC11 epithelial cell migration (Fig. 5G). Conversely, we found that the addition of AGEs directly to the tumor-derived Met1 epithelial cells led to a significant increase in epithelial cell migration (Additional file 4: Figure s4).

### AGE-stimulated activation of fibroblasts is RAGE-dependent

We previously demonstrated the expression of RAGE in normal mouse mammary fibroblasts. Since RAGE is a major receptor to the AGE ligand, we wanted to determine if fibroblast expression of RAGE is required for the AGE-mediated effects we observed on epithelial cell migration and invasion. Therefore, we isolated fibroblasts from the mammary glands of RAGE-/- and matched RAGE + / + littermates. Transwell migration and invasion co-culture assays were then conducted using Met1 cells as described. We found that the AGE-mediated increase in epithelial cell migration and invasion was ablated when RAGE-/- fibroblasts were used as the stimulating cells (Figs. 5G, H). Of note, a small but significant decrease in both epithelial migration and invasion was observed in the Met1 cells co-cultured with the RAGE-/- fibroblasts when compared to the RAGE + / + fibroblasts in the absence of any dietary or exogenous AGE treatment (Fig. 5G, H – white bars). These data suggest that mammary fibroblasts are a critical target for dietary AGEs and that RAGE is required for the observed effects, which in this case has been revealed through the use of a mammary gland model.

## Discussion

In order to address major health problems, it is critically important to identify environmental factors that contribute to disease in order to reduce or mitigate these influences. Diet, exercise, alcohol consumption, and tobacco use are all major environmental factors that contribute to obesity, diabetes, heart disease, hepatic disease, and cancer [45]. Programs targeting excessive tobacco and alcohol use have been effective health interventions [46, 47]. The role of diet in the development of obesity, diabetes and heart disease is well established, and there has been considerable research regarding the influences of diet upon cancer risk, progression and therapeutic resistance. Many studies have focused upon the presence of sugars and fats as primary drivers of disease due to their high caloric content. Energy-rich diets can provide excess calories resulting in the need to store excess energy in the body resulting in increased adiposity or other storage syndromes. This primary focus upon sugar as an energy source alone has obscured other potential impacts of sugar on tissues and cellular physiology, Furthermore, the manner in which foods are prepared can also have a major impact upon the influence that diet has on the body and health. One of these less well appreciated dietary components are AGEs.

AGEs result from non-enzymatic glycoxidation reactions that result in the modification of proteins, lipids and nucleic acids that can modify the structure or reactivity of these molecules, resulting in altered cell and tissue physiology. While some AGEs are produced within a cell or tissue that is provided high concentrations of sugars (endogenous AGEs), they can also be produced by cooking or grilled foods and thus are presented to cells and tissues as exogenous AGEs. Perhaps the most recognizable production of AGEs is the appearance of Amadori products when browning meats, which are desirable due to the flavors they produce. The AGE carboxymethyl lysine, or CML, is also added to some processed foods to improve taste and appearance. The downside of AGEs is that they can modify critical biomolecules and alter their structure and function. In addition, through binding to their cognate receptor RAGE, they can activate inflammatory pathways, and induce cell death (for a review see [5]).

In this study we have used rodent chow with a high sugar content as a low AGE diet and then autoclaved this chow to produce a high AGE diet; both of these diets were compared to regular rodent chow which has a high starch content rather than a high sugar content. Our previous studies have demonstrated that the AGE content of the regular diet is 517, the low AGE diet 15,504, and the high AGE diet to be 50,905 kU/1,000 kcal [27]. We also recently published an epidemiological study that showed the average daily CML-AGE consumption in humans was 6,105 ± 2,691 kU/1,000 kcal and ranged between 867 kU/1,000 kcal and 43,387 kU/1,000 kcal [48]. Therefore, our experimental diets are within the range of AGEs found to be consumed by humans.

To directly address the potential impact of a high AGE diet upon the development of a tissue system, we have chosen to use the mouse mammary gland as a model system. The development of the mammary gland can be studied postnatally and occurs in a timely and organized manner. We observed that provision of a high AGE diet between 3 and 12 weeks of age delayed ductal extension, increased ductal branching, and altered the structure of TEBs resulting in the appearance of highly proliferative structures with increased presence of macrophage and fibroblasts in an expanded ECM. These altered TEBs have many features of preneoplastic lesions suggesting that the high AGE diet may influence tumorigenesis. It is not clear whether the altered ductal structure reflects changes in the ECM, changes in cell proliferation, or both.

Gene expression studies of the different cell populations within the mammary gland have not demonstrated expression of RAGE, the receptor for AGEs [40]. It is particularly clear that mammary epithelial cells and their progenitor cells do not express RAGE. Because we observed an increase in fibroblasts within the ECM surrounding TEBs we explored whether RAGE was expressed in these cells and observed RAGE expression in fibroblasts isolated from mice fed either a regular or high AGE diet. Further characterization of these fibroblasts revealed that fibroblasts isolated from high AGE fed mice displayed markers of activated fibroblasts, such as cancer associated fibroblasts. Fibroblasts from high AGE mammary glands stimulated migration and invasion of mammary epithelial cells. Furthermore, the ability of AGE to stimulate fibroblast activation appeared to be RAGE-dependent. Studies by other investigators have suggested that changes in tissue histology that occur in patients as they progress from breast hyperplasia, through adenoma or in situ lesion to carcinoma can be specifically associated with the fibroblast compartment [49]. At this time, we cannot rule out a role for macrophages in these observations as we have not fully analyzed these cells and will be a focus of future studies.

The AGE-RAGE signaling pathway is a major orchestrator of the stromal microenvironment through paracrine signaling-mediated cellular crosstalk. One of the more striking phenotypes that we observed upon increased AGE consumption was increases in ECM surrounding both TEBs and atypical ductal structures that persisted into adulthood. TEBs are highly proliferative structures with defined head to tail polarity illustrated by the appearance of proliferating cells predominantly at the distal tip. Fibroblasts, macrophages and eosinophils localize at the neck and leading tip of the TEB to provide the environmental signals that guide epithelial proliferative growth. This is followed by cell differentiation into the luminal and basal layers that form the mature duct [22, 23]. AGE consumption led to the dysregulation of normal TEB function as illustrated by the increased recruitment of fibroblasts and inflammatory cells, proliferation of stroma surrounding the TEB, the lack of defined regional proliferation within the TEB and the increased TEB size. This is particularly evident by persistence of these features in mice of both 12 weeks and 7 months of age, a time at which TEBs are replaced by terminal end ducts. Of interest, is the potential impact of pubertal AGE exposure on mammographic density as a breast cancer risk factor given the increased abundance of stroma observed in mice fed the high AGE diet.

We posit, that the high AGE diet consumption is what leads to the persistence of these altered TEB-like structures as one of our more profound findings was the persistence of hyperproliferative TEB like structures and ducts into adulthood in these mice. Studies by other investigators demonstrated that provision of mice a high fat diet in the presence of a potent carcinogen (DMBA) resulted in similar structures and led to decreased breast tumor latency [50]. An important point to make here is the appearance of these pre-neoplastic lesions in our model is with high AGE diet alone and in the absence of a chemical carcinogen. Atypical hyperplasia in the breast is associated with a fourfold increase in the risk of developing breast cancer in later life [51]. Our recently published epidemiological studies in women detailing that increased consumption of dietary AGEs leads to increased risk of breast cancer among all women, increased risk of advanced stage and increased all-cause mortality in women with breast cancer [16, 48, 52] support our future plans to explore whether a high AGE diet accelerates mammary tumorigenesis in mouse models.

In this study, we use heat treatment of rodent chow that is high in sugar to drive the formation of AGEs and thereby simulate the presence of exogenous AGEs in the diet [53]. We believe that this is more reflective of the consumption of AGE-laden foods by humans, as it is well known that the rate of glycation in food is rapidly increased by the high dry heats and pressures applied during cooking (grilling, broiling and frying) processes and is dependent on sugar, fat and protein macronutrient content [54, 55]. However, it is possible that other components of the food that are heat labile may be affected by the heating process and impact directly on our experimental outcomes. An important outcome of our diet switch studies is that there exists a specific window during which the biggest impact of the high AGE diet was observed (3–7 weeks) suggesting that interventions targeted to this time period could have a major impact on reducing dietary risk.

## Conclusions

The role of nutrition and lifestyle on chronic diseases, including cancer, have become a central component to cancer prevention and control across the spectrum of the cancer care continuum. A greater understanding of the influence nutrition plays is a critical component to understanding the disease process. This study supports a direct cause and effect relationship between AGE exposure during puberty through diet, and sustained changes to mammary morphogenesis that may represent an early life event associated with increased breast cancer risk in later life. It serves to spotlight the importance of AGE biology and its role in increasing breast cancer risk in women and worse outcomes for women with breast cancer for future basic, translational and clinical studies in the field.

### Supplementary Information

**Additional file 1.** **Fig.** **S1** Individual mouse weight **(A)** and weekly food consumption **(B)** for each of the diet groups (n ≥ 4); regular (triangles), low AGE (circles) and high AGE (squares). **(C)** Quantitative ELISA of AGE levels in the serum of mice fed a regular, low AGE and high AGE diet (n ≥ 3) for 4 weeks compared to baseline levels. Values are mean ± SD.

**Additional file 2.** **Fig.** **S2** Quantitation of circulating Estradiol **(A)** and Progesterone **(B)** levels in 7- and 12-week old mice fed a regular (REG) or high AGE (HIGH) diet. **(C)** qPCR analysis of RANKL, AREG and IGF1 in fibroblasts isolated from mice fed a regular or high AGE diet. Values are mean ± SD.

**Additional file 3.** **Fig.** **S3** Representative images of ducts from 12-week old mice fed a high AGE diet demonstrating early stage, atypical ductal hyperplasia. **(B)** Percent distribution of abnormal structures within the proximal, middle and distal regions of the mammary gland.

**Additional file 4.** **Fig.** **S4** **(A)** Transwell invasion assay of Met1 cells co-cultured with either no fibroblasts or fibroblasts isolated from mice fed a regular (REG) or high AGE (HIGH) diet. Fibroblasts in the lower chamber were also either untreated or treated with AGE ex vivo. Transwell migration assay of **(B)** HC11 and **(C)** Met1 cells co-cultured with either no fibroblasts or fibroblasts isolated from mice fed a regular (REG) or high AGE (HIGH) diet. Epithelial cells in the upper chamber were also either untreated or treated with AGE ex vivo. *P < 0.05; **P < 0.01

## Data Availability

All data in our study are available upon reasonable request.

## Abbreviations

AGE: Advanced glycation end product
αSMA: Alpha Smooth Muscle Actin
CML: Nε-carboxy-methyl lysine
COL1A1: Alpha-1 subunit of the fibril-forming type I collagen
ECM: Extracellular matrix
FAP: Fibroblast activation protein
FBS: Fetal bovine serum
FSP1: Ferroptosis suppressor protein
H&E: Hematoxylin & Eosin
IHC: Immunohistochemistry
kU: Kilo units
MIN: Mammary intraepithelial neoplasia
RAGE: Receptor for AGE
TEB: Terminal end bud
TED: Terminal end duct
