# Supplementary figures and images for: Pubertal exposure to dietary advanced glycation end products disrupts ductal morphogenesis and induces atypical hyperplasia in the mammary gland
Source: Breast Cancer Res. 2023 Oct 6;25:118. doi: 10.1186/s13058-023-01714-4 (PMC10559657; doi:10.1186/s13058-023-01714-4)

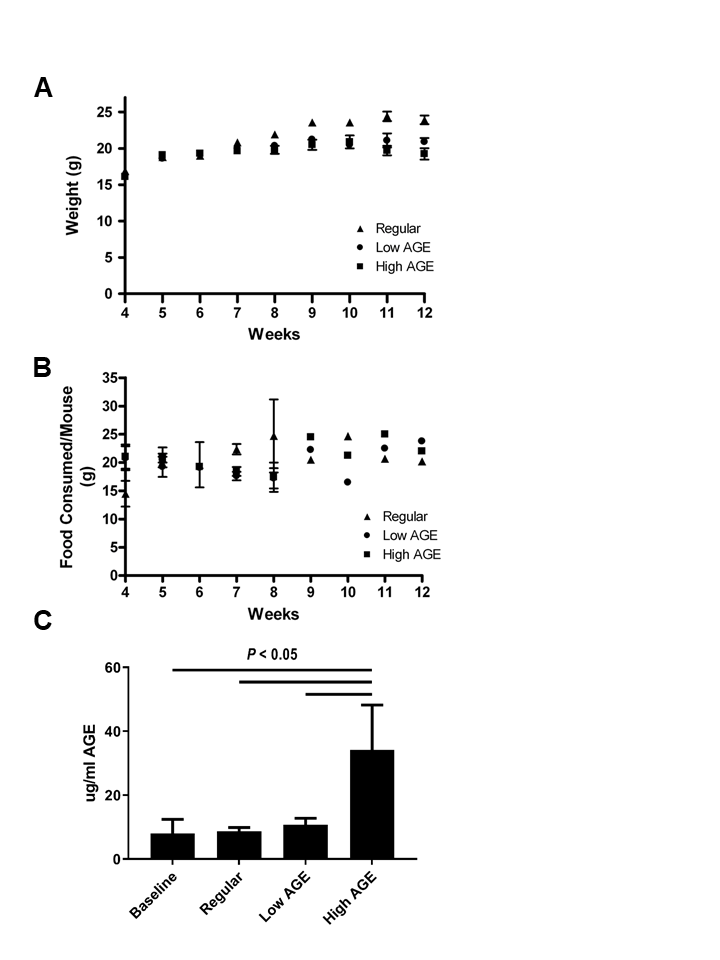

Supplement: Supplementary file 1 — Additional file 1. Fig. S1 Individual mouse weight (A) and weekly food consumption (B) for each of the diet groups (n ≥ 4); regular (triangles), low AGE (circles) and high AGE (squares). (C) Quantitative ELISA of AGE levels in the serum of mice fed a regular, low AGE and high AGE diet (n ≥ 3) for 4 weeks compared to baseline levels. Values are mean ± SD. [file 13058_2023_1714_MOESM1_ESM.tif]

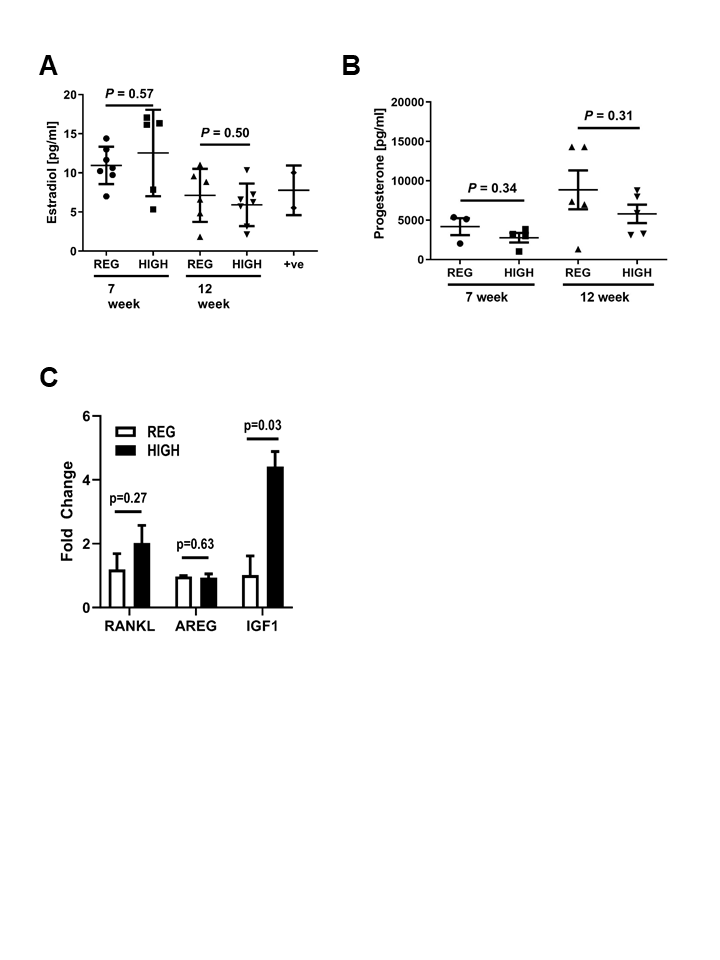

Supplement: Supplementary file 2 — Additional file 2. Fig. S2 Quantitation of circulating Estradiol (A) and Progesterone (B) levels in 7- and 12-week old mice fed a regular (REG) or high AGE (HIGH) diet. (C) qPCR analysis of RANKL, AREG and IGF1 in fibroblasts isolated from mice fed a regular or high AGE diet. Values are mean ± SD. [file 13058_2023_1714_MOESM2_ESM.tif]

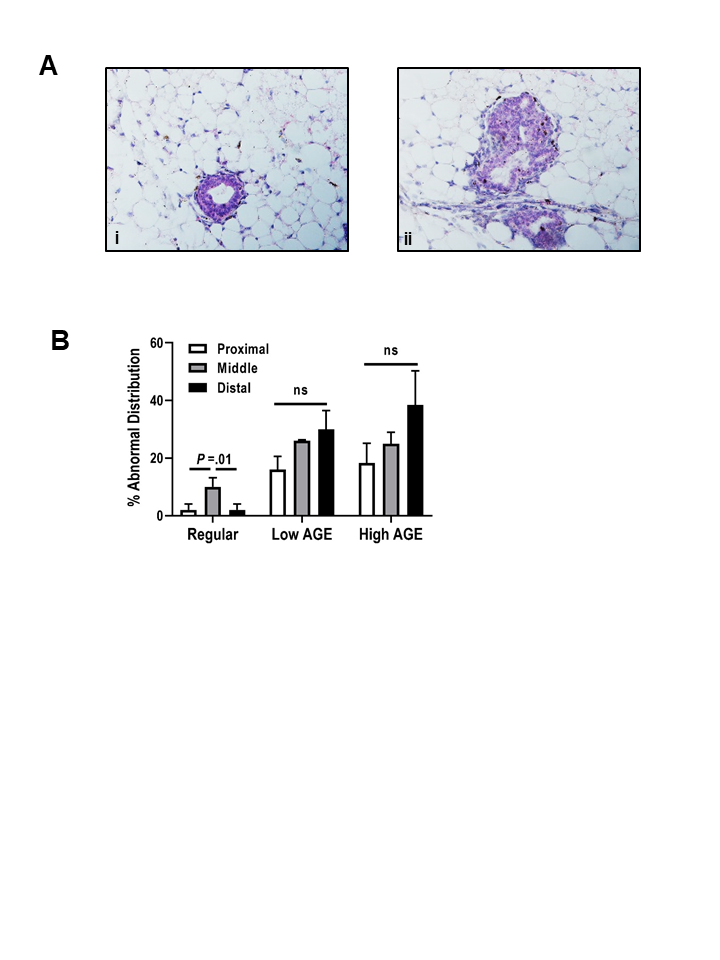

Supplement: Supplementary file 3 — Additional file 3. Fig. S3 Representative images of ducts from 12-week old mice fed a high AGE diet demonstrating early stage, atypical ductal hyperplasia. (B) Percent distribution of abnormal structures within the proximal, middle and distal regions of the mammary gland. [file 13058_2023_1714_MOESM3_ESM.tif]

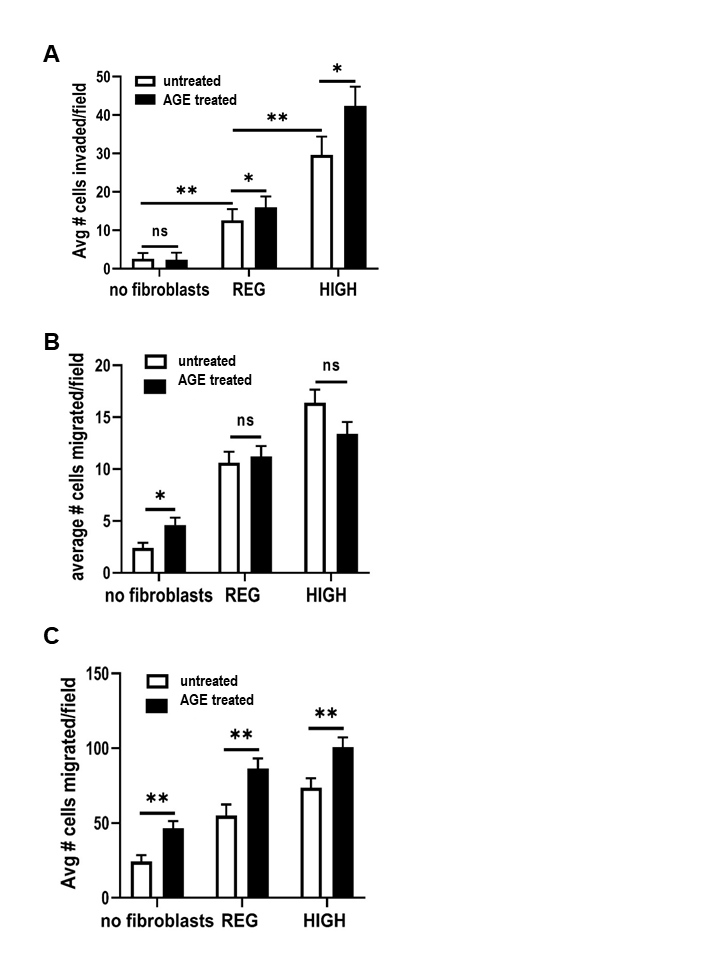

Supplement: Supplementary file 4 — Additional file 4. Fig. S4 (A) Transwell invasion assay of Met1 cells co-cultured with either no fibroblasts or fibroblasts isolated from mice fed a regular (REG) or high AGE (HIGH) diet. Fibroblasts in the lower chamber were also either untreated or treated with AGE ex vivo. Transwell migration assay of (B) HC11 and (C) Met1 cells co-cultured with either no fibroblasts or fibroblasts isolated from mice fed a regular (REG) or high AGE (HIGH) diet. Epithelial cells in the upper chamber were also either untreated or treated with AGE ex vivo. *P < 0.05; **P < 0.01 [file 13058_2023_1714_MOESM4_ESM.tif]
